# Subject- and Environment-Based Sensor Variability for Wearable Lower-Limb Assistive Devices

**DOI:** 10.3390/s19224887

**Published:** 2019-11-08

**Authors:** Nili E. Krausz, Blair H. Hu, Levi J. Hargrove

**Affiliations:** 1 Neural Engineering for Prosthetics and Orthotics Lab (NEPOL), Center of Bionic Medicine, Shirley Ryan AbilityLab (formerly RIC), Chicago, IL 60611, USA; blairhu2014@u.northwestern.edu (B.H.H.); l-hargrove@northwestern.edu (L.J.H.); 2 Biomedical Engineering Department, Northwestern University, Evanston, IL 60208, USA; 3 Physical Medicine and Rehabilitation Department, Northwestern University, Evanston, IL 60208, USA

**Keywords:** assistive robotics, prosthetics, sensor fusion, intention detection, computer vision, environmental sensing

## Abstract

Significant research effort has gone towards the development of powered lower limb prostheses that control power during gait. These devices use forward prediction based on electromyography (EMG), kinetics and kinematics to command the prosthesis which locomotion activity is desired. Unfortunately these predictions can have substantial errors, which can potentially lead to trips or falls. It is hypothesized that one reason for the significant prediction errors in the current control systems for powered lower-limb prostheses is due to the inter- and intra-subject variability of the data sources used for prediction. Environmental data, recorded from a depth sensor worn on a belt, should have less variability across trials and subjects as compared to kinetics, kinematics and EMG data, and thus its addition is proposed. The variability of each data source was analyzed, once normalized, to determine the intra-activity and intra-subject variability for each sensor modality. Then measures of separability, repeatability, clustering and overall desirability were computed. Results showed that combining Vision, EMG, IMU (inertial measurement unit), and Goniometer features yielded the best separability, repeatability, clustering and desirability across subjects and activities. This will likely be useful for future application in a forward predictor, which will incorporate Vision-based environmental data into a forward predictor for powered lower-limb prosthesis and exoskeletons.

## 1. Introduction

State-of-the-art control systems for powered lower-limb prostheses use hierarchical control systems and are often grouped into high-, mid- and low-level control categories [1]. Low-level control approaches use conventional engineering principles to compute current, position, or torque signals. Mid-level controllers generate reference trajectories that correspond to joint kinematics or kinetics and are typically implemented using state machines, where each state corresponds to a locomotion activity-specific controller [2] although generalized controllers have also shown promise [3,4,5]. High-level control often uses machine learning to predict the upcoming activity that the user is intending to perform [6]. Signal sources that are useful for predicting intent include—electromyographic (EMG) signals from within the prosthetic socket [7], or signals from sensors embedded into the construction of the prosthesis, often referred to as mechanical sensors [8]. These overall control approaches have allowed for real-time ambulation across a variety of terrains for several amputees [9].

While this approach is promising, misclassification errors still occur and can have negative consequences (e.g., falls). We hypothesize that one cause of prediction errors is the inter- and intra-subject variability of the data sources used for prediction. Subjects may walk with slightly different gait patterns from step to step or day to day and between subjects there may also be substantial differences in gait patterns, all of which will lead to inherent variability in the sensor signals used for forward prediction. To this end we have proposed the development of a sensor fusion-based forward predictor that incorporates environmental data, recorded from a wearable vision-based sensor, in addition to subject-based EMG and mechanical sensors. We hypothesize that this data should have lower variability across trials and subjects as compared to kinetic, kinematic and EMG data.

Computer vision has been successfully applied in several applications in recent years from self-driving cars to robotic vacuum cleaners. Computer vision has also been proposed for improving the control of both upper and lower-limb powered prostheses [10,11]. Our group has also presented a stair ascent recognition system, which was developed as an attempt to eliminate high-level control errors due to misclassification of stair ascent, which have been shown to be critical and lead to major perturbations to the user [12]. We built on past work to develop a novel segmentation algorithm that locates stairs in the environment and produces a series of secondary features that could be used to estimate user intent. Preliminary testing of our algorithm was promising, with a step accuracy of 100% during an online walking test recorded at 5.15 fps [13]. We have since expanded the developed algorithm to include ramps and stair descent [14]. Additionally, we presented work to use depth sensing for gait segmentation, in addition to inertial sensors, to improve timing of steps, which ultimately should improve forward prediction as well [15]. Recently, several other research groups have presented preliminary results for the development of other predictive systems for powered prostheses or exoskeletons that use environmental sensors, including [11,16,17,18,19,20,21,22,23,24,25]. These works are important and show the utility of using environmental based sensors for improving high-level control of assistive devices.

However, to our knowledge, there has yet to be a study that seeks to understand the underlying differences between environmental and subject-based sensor modalities. We aimed to consider the data separately from the specific algorithm or wearable device being used. Therefore, in this work we present a device-agnostic, feature-driven approach and analysis of the variability within and between subjects and activities for each sensor modality. Though the work presented herein is not dependent on a specific control system, in our future work we aim to extend this towards the development of robust sensor-fusion based forward predictor that functions accurately across trials, days, subjects and potentially even wearable devices, which would help enable the translation of wearable assistive devices out of the lab.

## 2. Methods

### 2.1. Data Collection

**Figure 1:**
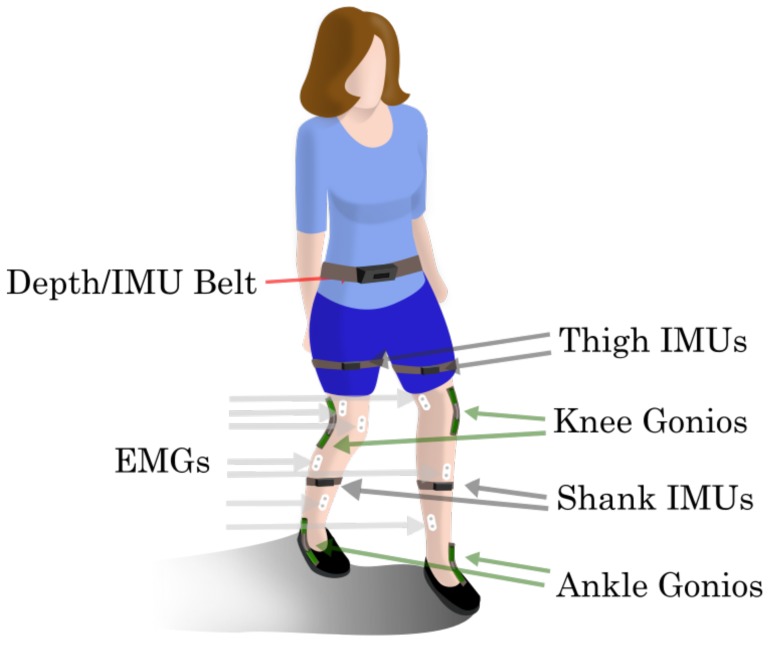
Experiment Setup. Subjects had bilateral thigh and shank IMUs, knee and ankle goniometers and EMG sensors on 7 leg muscles ( tibialis anterior (TA), medial gastrocnemius (MG), soleus (SOL), vastus lateralis (VL), rectus femoris (RF), biceps femoris (BF) and semitendinosus (ST)) and a belt containing a vision sensor (Camboard Pico Flexx [28]) and a 6 axis IMU.

The data analyzed in this paper was previously described in Reference [26] (and made freely accessible in Reference [27]), with the exception of the data, setup and processing of the sensors in the Vision/IMU belt, thus only a short summary of the data collection procedure is included here. The Northwestern University Institutional Review Board approved this study and written consent was obtained from 10 able-bodied subjects (7 male, 3 female; 23–29 years, 160–193 cm, 54–95 kg) who completed the experiment. Subjects were instrumented bilaterally with bilateral 14 EMG sensors, 4 Goniometers, 4 IMUs, plus a Depth/IMU belt, all of which were tethered to our data acquisition system. Figure 1 shows an illustration of the complete sensorization.

During the experiment, subjects completed 25 circuits consisting of level ground walking (LW), ascending/descending a ramp with a 10${}^{\circ }$ slope (RA/RD) and ascending/descending a four-step staircase (SA/SD) step-over-step. Subjects walked at their own pace and the experimenter then labeled the true locomotor intent of the subject using a custom GUI. Heel contact and toe off gait events for each leg were later identified based on shank velocity.

In addition to the EMG, IMUs and goniometers that were described in [26], subjects wore a belt containing a single time-of-flight (ToF) vision sensor (Camboard Pico Flexx [28]) and a 6 axis IMU aligned and oriented at a slight downward angle. This sensor was synchronized with the other sensors using a custom Matlab script that was triggered by our proprietary data acquisition system. In this experiment the vision sensor was operated at a frame rate of 5 fps, with a max exposure time of 2000 μs and a general range of 1–4 m, though it was possible to see objects closer than 1 m due to the angle and positioning of the sensor. This setting was selected experimentally, as this was appropriate for the range required for recognizing environmental changes. Additionally, this setting was selected as our preliminary testing has shown it to be sufficient for use with a powered knee-ankle prosthesis without causing latency that would result in trips, stumbles, or falls. The IMU was used to re-orient the vision-based point clouds from a camera-reference frame to a global-reference frame. This was done using a standard Euler-angle approach. A complementary filter was used to reduce drift and improve the orientation estimate for the waist sensor. This IMU was disconnected during the experimental session for 2 subjects, thus the depth features were not able to automatically be reoriented and thus these subjects were excluded from the analysis, thus ultimately the analysis only includes data from 8 subjects. Additionally, prior to feature extraction the vision-based point clouds were denoised using a simple nearest neighbor approach that eliminates outliers.

### 2.2. Feature Extraction

To quantify the variability of the different sensor modalities we considered a feature-driven approach. In this method we selected features to represent the data, normalized the features (as described in Section 2.3 and then compared the variability of each feature set for each subject and between subjects. These features could later also be used for building a machine learning-based intent recognition system, though the variability analysis itself is classifier agnostic and will instead provide insight into the underlying structure of the data. The subset of features with the lowest inter and intra-subject variability were identified and will eventually be used for development of a robust forward predictor.

#### 2.2.1. EMG, IMU and Goniometer Features

Immediately prior to each heel contact or toe off gait event identified within each trial, a 300 ms analysis window was segmented from all data channels (one window/event). Each window of data was then used to extract features from the EMG, Goniometer and IMU sensors. The features that were selected for extraction were those that have been used previously in the literature for intent recognition for powered knee-ankle prostheses. For each IMU and goniometer channel, six features were extracted per window including the mean, standard deviation, maximum, minimum, initial and final values [29]. The EMG features meanwhile included the mean absolute value (MAV), waveform length, number of zero crossings and slope sign changes and the coefficients of a sixth-order autoregressive model [30,31]. Bilaterally there were 14 EMG sensors (one channel per sensor), 4 goniometers (1 channel per sensor) and 5 IMUs (6 channels per sensor). Thus there were a total of 140 EMG features, 24 Goniometer features and 180 IMU features per gait event.

#### 2.2.2. Vision-Based Features

**Figure 2:**
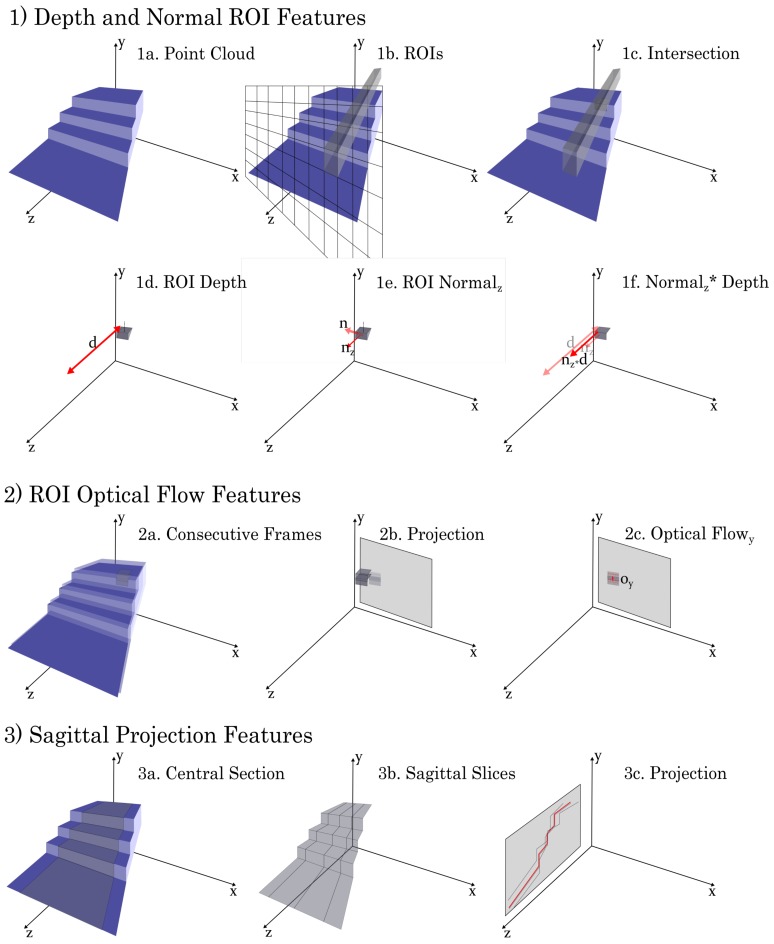
Vision-Based Features. Features were extracted from point cloud frames just prior to gait events. For each frame (**1a**) a series of features was extracted for comparison as follows—(1) The field of view was segmented into a 20 × 20 ROI grid (**1b**) and the points within each region of interest (ROI) were fit with a RANSAC (RAndom SAmpling Consensus [32]) planar model. The mean distance from the sensor to the plane in the z dimension was defined as the Depth feature (**1c**). The y and z components of the normal vector were defined as the Normal features (**1d**). These two features were multiplied together to combine depth and orientation information into a single Normal × Depth feature (**1f**). (2) Two consecutive frames (**2a**) were projected onto a 2D plane (**2b**) and motion across frames was computed a dense Farneback optical flow method [33], with the optical flow component in the x- and y- directions averaged within each ROI and defined as the Optical features (**2c**). (3) The outer third of each frame was filtered out to help minimize effects of occlusions and objects within the field of view (**3a**) and then all points were projected into the sagittal plane (**3c**), with the mean height of each position in the z-direction being defined as the Sagittal feature (**3c**).

Vision-based point clouds were acquired at every frame, with dimensionality of 171 × 224 × 3, representing the x, y and z dimension of each pixel. The frame just prior to each gait event was used to extract features for variability comparison. The features chosen were intended to be representative of the general content of the point cloud and these were inspired by features that have been previously used in literature. Once features were extracted they were vectorized to aide comparison with the other sensor modalities. From each point cloud the series of features extracted for comparison was as follows:

1. *Depth and Normal ROI features*: Each frame was segmented into a 20 × 20 grid of regions of interest (ROIs) and all points within each ROI were then fit with a RANSAC [32] planar model (see Figure 2(1a–c)). Three features were then defined for each of these ROIs:
  (a) *Depth*: The mean distance from the origin of the sensor to the plane in the x-, y- and z-dimension were defined as Depth features (Figure 2(1d) shows the z-feature). These features essentially provided the projection of the depth in the frontal and sagittal planes and these were selected to provide average depth context for field of view.
  (b) *Normal*: The normal vector of the plane provides orientation context for each ROI. In particular, the magnitude of the y- and z-components of the normal vector of the plane were computed and defined as the Normal features (Figure 2(1e) shows the z- feature). These features were selected to help provide orientation context that might be missing from the depth features alone.
  (c) *Norm x Depth*: The Depth and Normal features for each ROI were then combined, using a simple scalar multiplication, into a single Norm x Depth feature (Figure 2(1f)). This feature was chosen as an attempt to represent the depth and orientation content efficiently in a single feature.
2. *Optical Flow features*: Motion across frames was computed both as the simple difference between frames and using a dense Farneback optical flow method [33]. In particular, the frontal plane projection of the depth was used for the optical flow estimation. The optical flow components in the x- and y- directions were each averaged within each ROI of a 20 × 20 grid and defined as the Optical features (Figure 2(2c)). These features were selected as a means of providing context about the motion of the environment relative to the subject.
3. *Sagittal Projection features*: The point cloud was filtered so that only the central two-thirds were considered, to help minimize the effect of occlusions and random objects within the field of view. Then all points were projected into the sagittal plane and the mean height of each position along the z-axis was then defined as the Sagittal feature (Figure 2(3c)). This feature was selected as a means of encoding context about the overall shape of the environment (particularly the height which was less integrated into other features).

The best performing Vision-based features were compared to the IMU, Goniometer, EMG features and merely referred to as the “Vision” feature set throughout the remainder of the analysis.

### 2.3. Normalization

**Figure 3:**
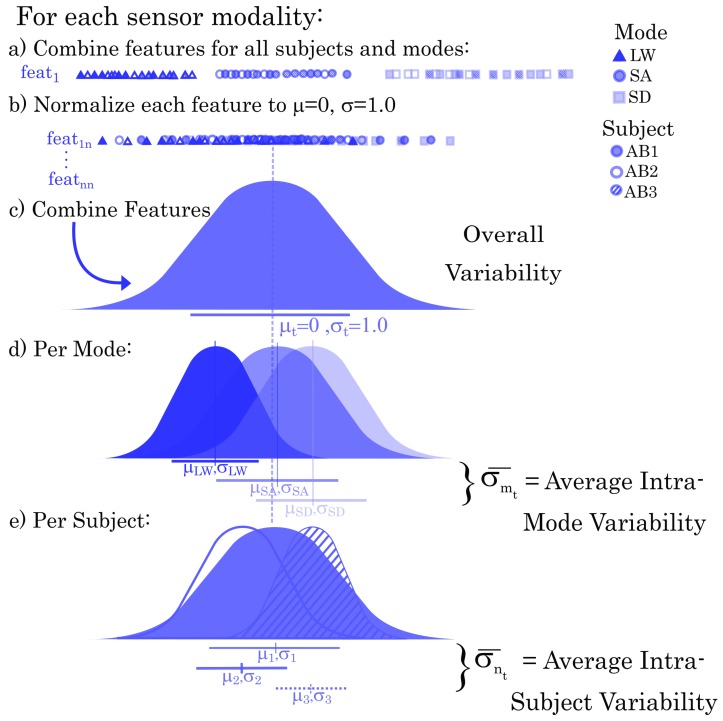
Normalization procedure for each sensor modality. This example shows 3 subjects and activities, simply for ease of illustration, but in reality all subjects and activities are included in the normalization. For each feature in the sensor feature set all subjects and activities are combined, as in (**a**). Then each feature is normalized (**b**). All features are then combined into the overall distribution (**c**) and the distributions for each activity (**d**) and each subject (**e**) are then extracted. From here the overall average intra-activity and intra-subject variabilities can be computed using a simple averaging.

The goal of this work was to compare sensor modalities with differing units and ranges, thus it was important to normalize features to allow for direct comparisons. As the ultimate comparisons would be highly dependent on the normalization procedure used we very carefully considered how best to normalize our data to ensure that the results were meaningful. Features from all subjects and activities were combined prior to normalization, which allowed for an overall understanding of how variability differs within and between activities and subjects for each sensor modality. We considered the variability for heel contact (HC) and toe off (TO) gait events separately. For each gait event samples for all subjects and modes were combined (Figure 3a) and then each feature was normalized to have a mean of 0 and a standard deviation of 1 (see Figure 3b,c). The mean and standard deviation of all features during events of each mode were then computed. Thus, we able to obtain the intra-mode variability, $\sigma_{m}$, for each locomotion mode, *m* (Figure 3d). Similarly we can acquire the intra-subject variability, $\sigma_{n}$, for each subject, *n*, (Figure 3e). The average intra-mode and intra-subject variabilities, $\bar{\sigma_{m_{t}}}$ and $\bar{\sigma_{n_{t}}}$ respectively, can then be compared across sensor modalities.

However, as there is a dimensionality mismatch between feature sets it may be necessary to consider more extensive analysis with scaling-invariant metrics to more conclusively determine the within activity and subject variability.

### 2.4. Dimensionality Reduction

In addition to the differences between scales for the feature sets there was also concern that the different number of features would affect the variability analysis. Therefore, once normalization was completed, dimensionality reduction was performed so that comparisons between data sets with different dimensions could be made. In particular a Linear discriminant analysis (LDA) dimensionality reduction procedure was performed so as to maintain class separability in the reduced features. The four output LDA components were then used to analyze each sensor modality. Scatter plots and density estimations are shown for the LDA components that were analyzed are shown in the Dimensionality Reduction results section.

### 2.5. Sensor Modality Analysis

In [34], Bunderson and Kuiken proposed three measures for characterizing an EMG feature set across classes and trials. These measures are designed to provide scale-independent metrics for analysis across feature types and classes. In our analysis we treat each subject as an independent “trial” and each activity as a separate “class” and use the proposed metrics to compare between sensor modalities. Each metric was computed five times and the average value of each metric for each feature set was reported.

#### 2.5.1. Repeatability Index

The Repeatability Index (RI) provides an idea of how a single individual represents the data from one “trial” to the next, or in our case subject. RI provides an estimate of how much similarity there is between *k* subjects across *j* activities, with lower RI values signifying greater repeatability or overlap between subjects. RI is calculated for each subject using Equation (1), where $S_{j}$ is the data covariance for activity class *j*:

(1) $$\;RI=\frac{1}{m}\sum_{j=1}^{m}\left(\frac{1}{n}\sum_{k=1}^{n}\frac{1}{2}\sqrt{{\left(\mu_{j}-\mu_{k}\right)}^{T}S_{j}^{-1}\left(\mu_{j}-\mu_{k}\right)}\right)\;$$

The RI is then averaged across all *n* subjects to allow for comparison between sensor modalities. Computed RI results are discussed in Section 3.3.1.

#### 2.5.2. Separability Index

The Separability Index (SI) measures interclass distance. In particular, 1/2 the distance from a given activity class *j* to the nearest class *i* is computed and this value is averaged across all *m* classes. Higher SI values are preferred as they signify greater distance between classes. Equation (2) is used to compute the SI:

(2) $$\;SI=\frac{1}{m}\sum_{j=1}^{m}\left(\min_{i=1,\ldots m}\frac{1}{2}\sqrt{{\left(\mu_{j}-\mu_{i}\right)}^{T}S_{j}^{-1}\left(\mu_{j}-\mu_{i}\right)}\right)\;$$

#### 2.5.3. Mean Semi-Principal Axes

As separability can be related both to more tightly clustered classes or to greater distance between classes in feature space, we also considered the shape of the classes, using the third metric presented by Bunderson and Kuiken [34] which is a measure of the hyperellipsoid size of each class. In our case we used Principal Component Analysis (PCA) to find the principal axes for each class and measured the geometric mean of each semi-principal axis (MSA). We have 4 principal axes, corresponding to our feature dimensionality *f* of 4. The MSA is then averaged across all classes, to allow for comparisons between sensor modalities. Lower MSA values are preferable as this is a measure of the overall variability within each class. Equation (3) is used to compute the MSA:

(3) $$\;MSA=\frac{1}{m}\sum_{j=1}^{m}\left({\left(\prod_{f=1}^{4}a_{jf}\right)}^{1/4}\right)\;$$

#### 2.5.4. Desirability Score

For selection of the ideal feature set or combination we combined the SI, RI and MSA to compute a desirability score (DS) that could be compared across sensor modalities. Desirability is computed using Equation (4), as follows:

(4) $$\;DS=\frac{SI}{MSA*RI}\;$$

Since higher values of SI and lower values of both RI and MSA are preferred, higher DS values are preferred.

## 3. Results

A consistent color scheme is used throughout the results figures to help keep track of the specific sensor modality being shown, with colors representing feature sets as follows: green = IMU, blue = Goniometer, cyan = EMG, purple = Vision. Light shades are used for sensor-specific feature sets and dark shades are used for combination feature sets. Additionally, for all figures the HC results are shown and discussed for simplicity, however TO results are comparable.

### 3.1. Normalization

**Figure 4:**
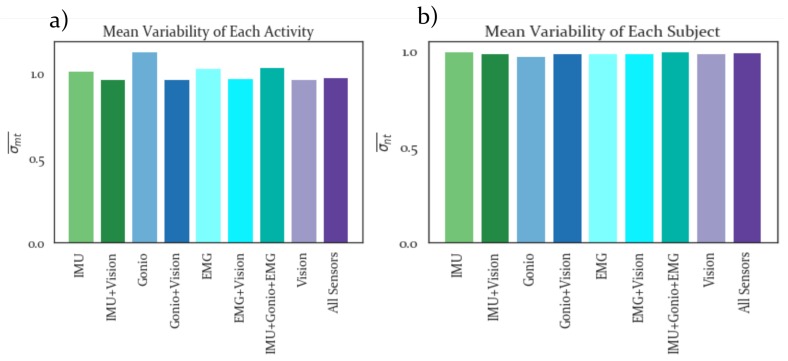
Normalization Results. (**a**) Intra-activity and (**b**) intra-subject variability computed from Normalized feature sets. Lower variability is preferrable, with all feature sets that include vision having lower variability than those that do not include vision. These results may be affected by the differences in dimensionality between feature sets, however.

Figure 4a,b show average intra-activity and intra-subject variability results, respectively. In both cases lower values are preferable, as this signifies greater consistency between trials for a given subject or activity. The feature sets that include Vision have slightly lower intra-activity variability than those that do not include Vision and all feature sets have negligible differences in intra-subject variability.

### 3.2. Dimensionality Reduction

**Figure 5:**
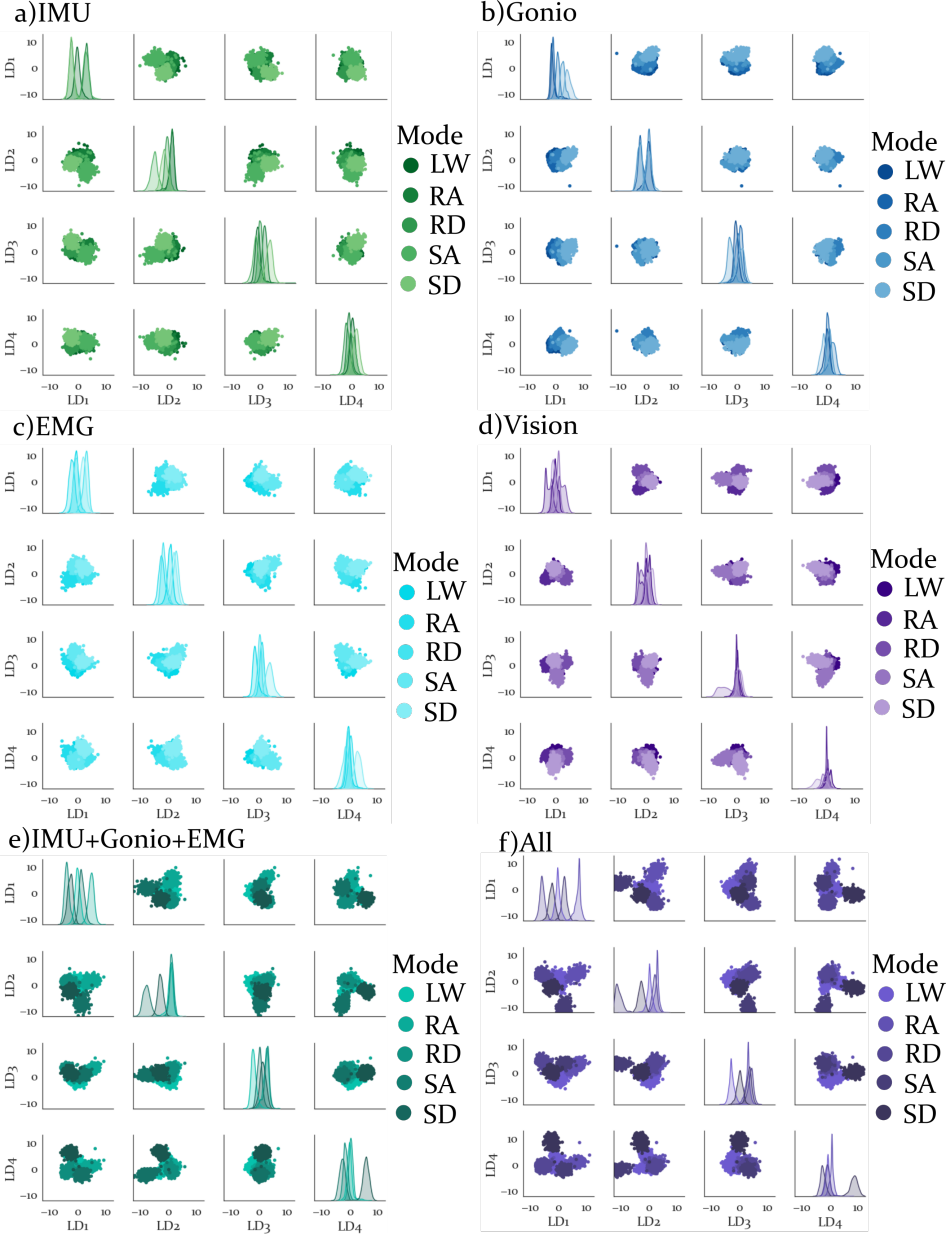
Dimensionality Reduction results for each sensor modality. LDA components 1-4 for (**a**) IMU features, (**b**) Goniometer features, (**c**) EMG features, (**d**) Vision features, (**e**)IMU, Goniometer and EMG features combined and (**f**) All features combined. For each sensor modality, subplots along the diagonal show estimated density functions for each activity and LDA component. Non-diagonal subplots show the projection of the feature space onto any two LDA components.

Results from the dimensionality reduction are shown in Figure 5. Each sensor modality is represented by a 4 × 4 series of subplots. Smoothed distributions of the LDA components for each activity are shown along the diagonal subplots. On the non-diagonal subplots each LDA component is plotted against the other LDA components. Varying shades are used to represent different activities for each sensor modality. Based on visual inspection alone it appears that the plot with all features combined has the most clearly separable distributions, while simultaneously having the largest LDA components relative to any of the sensor-specific plots. Thus it is expected that the SI and MSA will both be highest for all features combined. It is difficult to say based on this plot alone whether the RI is anticipated to be higher or lower than the individual sensors alone.

### 3.3. Sensor Modality Analysis

#### 3.3.1. Repeatability Index

**Figure 6:**
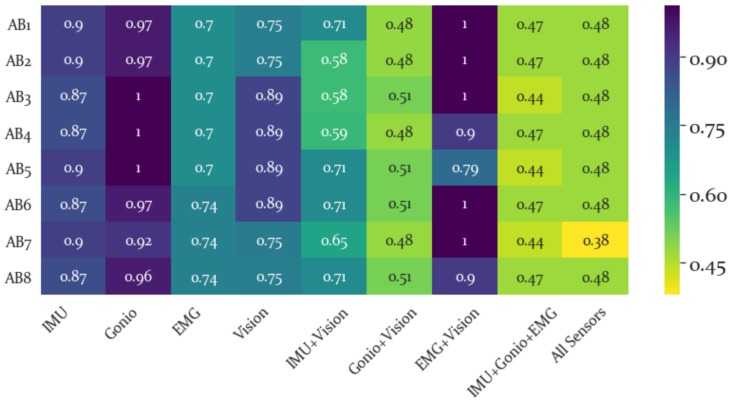
Repeatability Index for each subject and sensor modality. Inter-subject differences can be indentified using this heatmap. For instance, AB4 has the highest RI for the EMG+Vision feature set. This indicates that this subject was least repeatable, particularly in these sensor modalities.

The RI for each subject and sensor modality is shown as a heatmap in Figure 6. Lower (or brighter) values are more desirable and higher (or darker) values are less desirable. The inter-subject differences in repeatability can be evaluated to understand how each sensor modality varies between subjects. For instance, AB7 had the lowest RI for the “All” feature set, indicating that this subject’s combined features represented the space well for other subjects.

**Figure 7:**
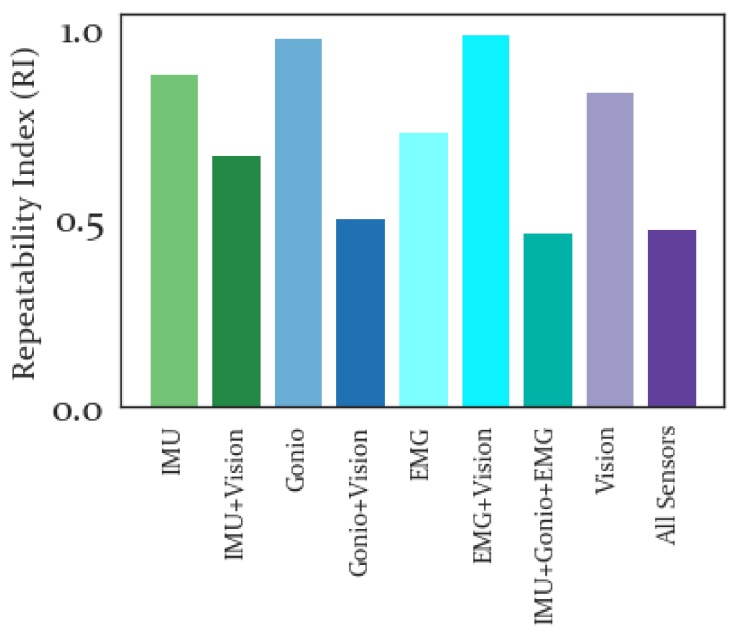
Repeatability Index averaged across all subjects. Lower values indicate greater repeatability from one subject to the others. Of the sensor-specific feature sets, Vision was most repeatable and Goniometer was least repeatable.

The RI for each sensor modality, averaged across all subjects, is shown in Figure 7. The lowest RI was produced by all features combined, though the Gonio+Vision and IMU+Gonio+EMG indices were similarly low. This indicates that these combined feature sets represent the space across subjects better than the other sensor modalities and feature sets.

#### 3.3.2. Separability Index

**Figure 8:**
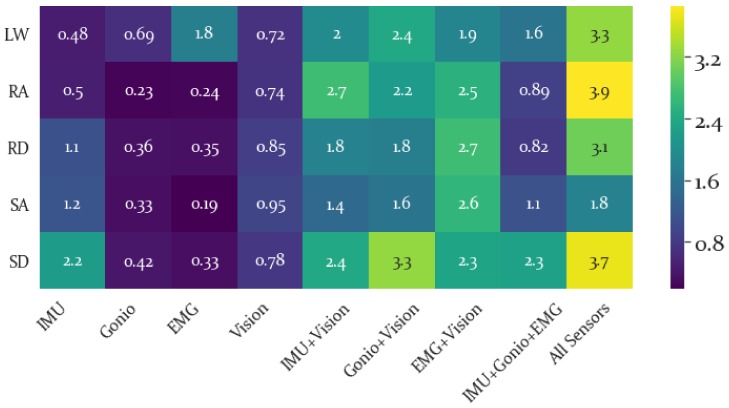
Separability Index for Each Activity. Higher (brighter) values are preferable, indicating greater separability for a given activity and feature set. The greatest separability was found for ramp ascent, RA, when combining all sensor modalities. Using this heatmap it is possible to identify the specific sensor modality that is most separable for a given locomotion activity, such as noting that EMG feature sets had highest LW separability, while the IMU had highest SI for RD, SA and SD. Similarly, the most separable combinations of sensors for a given activity can be identified using this heatmap.

The SI for each locomotion activity and sensor modality is presented in a heatmap in Figure 8. From here it is possible to evaluate the locomotion activities that are most separable and which sensor modality is best able to separate a given locomotion activity within the LDA feature space.

**Figure 9:**
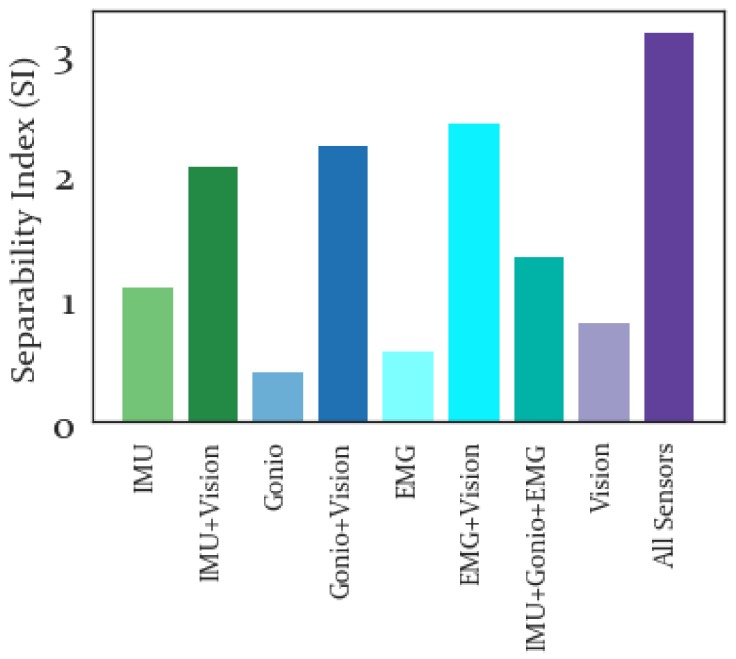
Average Separability Index for All Sensor Modalities. Higher values indicate greater separability of locomotion activities using a given sensor set. The sensor-specific feature sets had lower SI than the combined feature sets, with all features combined having a significantly higher SI than any other feature set.

The average SI for each sensor modality is shown in Figure 9 and higher values of SI are preferable. Of the individual sensors the best separability was produced by the IMU features and the worst separability was produced by the Goniometer features. Adding Vision features to the IMU, Goniometer, or EMG feature sets resulted in improved separability.

#### 3.3.3. Mean Semi-Principal Axis

**Figure 10:**
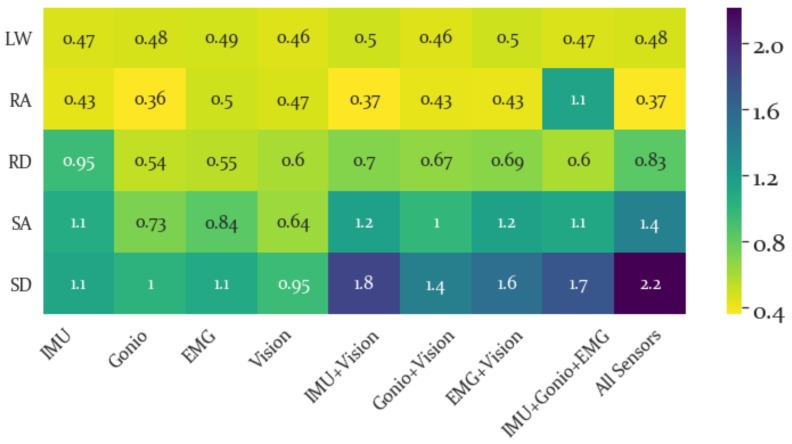
Mean Semi-Principal Axis for Each Activity. Lower (brighter) values are preferable, as this signifies tighter clustering within a given activity class. This heatmap can provide context for how well clustered features are for a given activity class. For instance, generally SD had the highest (and poorest) MSA, while RA had the lowest (and best) MSA values.

The MSA results for each activity class and sensor modality are shown in Figure 10. These results show that SD was the least tightly clustered activity, with highest values in general. RA meanwhile had lowest MSA in general and this was also the case when all feature sets were combined.

**Figure 11:**
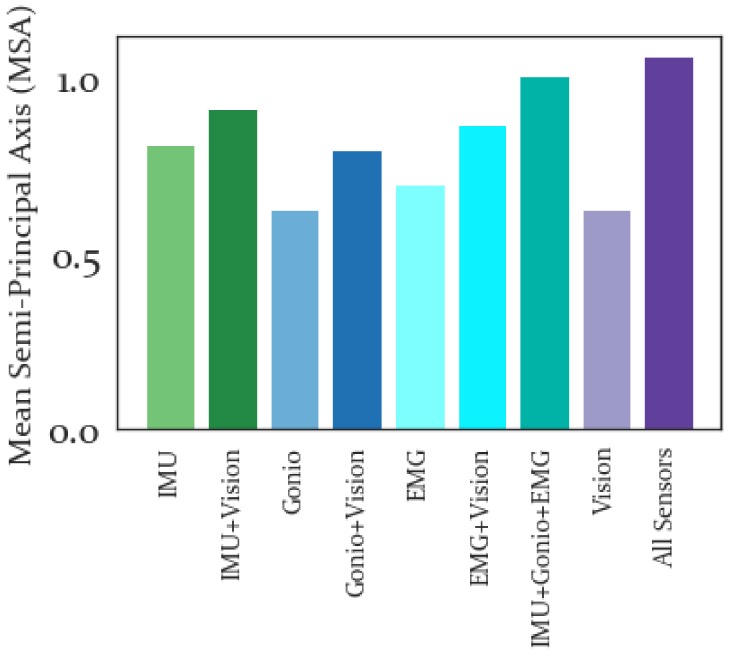
Average Mean Semi-Principal Axis Results. Low values of MSA are better. All features combined performed only slightly worse than the IMU+Gonio+EMG sensor-specific feature sets. The largest MSA was produced by all features combined.

The average MSA for each feature set is shown in Figure 11. MSA represents the size of the activity-specific clusters, so lower values are better. The worst MSA result for the sensor-specific features was produced by the IMU feature set and the overall worst MSA performance was produced by all features combined. In general, the combination feature sets performed worse than the sensor-specific feature sets. However, the feature set with all sensors combined performed only marginally worse than the IMU+Gonio+EMG sensor set.

#### 3.3.4. Desirability Score

**Figure 12:**
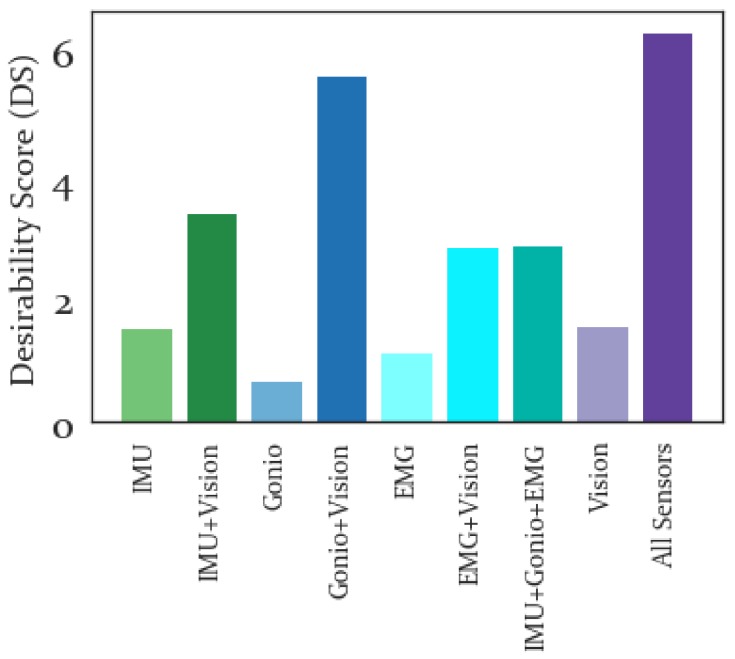
Desirability Score for each sensor modality. Higher values of DS are preferred, with all features combined performing substantially better than any of the sensor-specific or combined feature sets.

The DS was computed for each feature set, with results shown in Figure 12, and in general the combined feature sets performed better than the sensor-specific feature sets, with the best performance produced by combining all features.

## 4. Discussion

Data was collected for able-bodied subjects across 5 locomotion activities, wearing a number of wearable sensors, as described in [27] and the Data Collection section of this manuscript. Features were extracted from worn IMUs, goniometers, EMGs and a wearable Vision/IMU sensor belt. These features were then normalized and analyzed to provide an understanding of the variability of each sensor modality across activities and subjects. Results were considered to determine the feature set that performed best in these specific ambulation tasks.

### 4.1. Result Implications

All feature sets that included the Vision features had lower intra-activity variabilities than those that only included IMU, Gonio or EMG features, based on the normalization results, see Figure 4. To correct for differences in dimensionality between sensor modalities it was necessary to perform dimensionality reduction. The results in Figure 5 demonstrate that the LDA dimensionality reduction was able to preserve clustering between activities for each sensor modality with only four components. Additionally, visual inspection of these results showed that the feature set with all sensor modalities combined had greatest separability, which was confirmed by the SI results.

Average RI was found to be lowest for all features combined and highest for the EMG+Vision feature set(see Figure 7). However, for the IMU and Goniometers the RI improved with the addition of Vision. Importantly this measure provides an estimate of the generalizability of a given feature set across subjects and thus all features combined was quite repeatable across subjects (see Figure 6). Importantly, adding Vision to the IMU and Goniometer features improved their repeatability. Thus we anticipate that incorporating Vision-based features into a forward predictor can make the development of a general, non subject-specific, forward predictor more feasible and stable.

The average SI results are shown in Figure 9 and the best separability by a large margin was produced by combining all feature modalities. Despite Vision features being second to IMU features in terms of highest SI, adding the Vision feature set increased the SI for IMU, Goniometer and EMG feature sets. This implies that though the Vision features themselves do not have the highest separability, they are able to increase overall separability when combined with other sensor features. In particular, the activity-specific SI results were shown in Figure 8 and the Vision features did not yield particularly high SI values for any activity. However, the highest SI values (excluding all features combined which was highest for all activities) for each activity were produced using a combination of the Vision features with other sensor modalities.

Meanwhile, the SI results show that the best individual sensor modality for differentiating stair ascent and descent and ramp descent was IMU, while EMG alone was best at differentiating level walking. A potential explanation for why EMG (and to some extent the Gonio features) performed well for level ground but not stair or ramp activities is that the muscle patterns for stair ascent likely are somewhat similar to those of ramp ascent and the muscle patterns of stair descent are likely not that different from those of ramp descent and level walking is the most distinct in terms of the general kinematics. While it is somewhat surprising that the Gonio features are not better at discriminating between stair and ramp activities, it is not surprising that the IMU performed well, given the differences between motion on stairs, ramps and level ground. Meanwhile, Vision produced fairly similar separability across activities, indicating that it is less sensitive to the specifics of the activity than to the general structure of the environment, which is intuitive. However, the addition of vision generally increased separability across the board and relative to the IMU+Gonio+EMG feature set adding vision yielded higher separability for all classes, though surprisingly the smallest change between these two conditions was for SA. Presumably by incorporating environmental data ambiguity between locomotion activities (when considering kinematics and kinetics alone) is resolved.

MSA results are shown in Figure 11. Lower MSA values signify tighter clustering and therefore are more desirable. This measure can be thought of as the intraclass variability. Combining all feature sets produces the lowest average MSA, relative to any other feature set combinations. Meanwhile, the IMU features had the highest sensor-specific average MSA value, implying that IMU features are less tightly clustered than EMG, Goniometer or Vision features across subjects. Additionally, if the activity-specific MSA results are considered (see Figure 10) it becomes clear that the activity that is clustered most poorly is SD, and the activity that is most tightly clustered is RA. The MSA primarily shows that features are most repeatable across subjects within a given activity for ramp ascent and level walking and less repeatable for the remaining activities. Potentially this is due to the challenges of controlling power dissipation or the precision needed for controlled, reciprocal stair ambulation or ramp descent.

Finally, the DS was computed for each feature set and the overall best performance is obtained when combining all feature sets. In particular, the Gonio+Vision feature set was the only feature set that even remotely approached the DS obtained by combining all features. This implies that a fusion of all four sensor modalities would be ideal for inclusion in a forward prediction system, to provide the best separability, repeatability, and clustering within and between activities. However, if reduced sensorization is required, the best performing combination were the Goniometers and Vision sensor. Surprisingly the IMU features did not perform as well as expected, often being outperformed by the Goniometers and even EMG features. Part of this effect may be due to the fact that only raw velocity and acceleration IMU signals were used for feature extraction, without orientation or angle computations implemented. Presumably if a device does not include a goniometer, a different sensor providing angular measurements would perform similarly, such as a potentiometer or encoder measurement at the joint, or potentially even an IMU estimating the orientation change of each joint. Any of these sensors ought to perform similarly to the goniometers, and would require less sensorization than the full combined sensor set. Therefore, we believe that the performance of these features is a good sign for the eventual implementation of this approach on a powered lower limb prosthesis or other wearable robotic device.

Robustness of each sensor modality across days and environmental conditions is another factor that is important for consideration in future to ensure that selected sensors are best suited for implementation in a wearable assistive device. In this case the sensor selected for use was a small ToF-based sensor, which was chosen to be complementary to the approaches used in our previous works [13,14] and to provide contextual data that could ultimately be implemented for not only accurate forward prediction, but also gait trajectory predictions, early planning of desired activities, and even potentially for simultaneous localization and mapping (SLAM) of select environments (though in this work the use of this sensor for forward prediction is primarily considered). However, there are a number of potential environmental sensors that could have been selected for use in this work, and a more extensive analysis of the best sensors and configurations for wearable assistive robotics is presented in our upcoming Review paper [35].

Further investigation of how to combining feature sets may help inform the design of a forward prediction system that has good separability between all desired activities. Though the sample size included herein is smaller than ideal, for this proof-of-concept study we used a previously published dataset to allow for validation and comparison with the results presented in that work. Additionally, the extensive sensorization time and experimenter demands were limiting factors in collecting a larger population size, and ultimately the goal of future work will be to validate these results with amputee subjects walking on an actual prosthesis which would have several sensors embedded, thus reducing the sensorization needs.

### 4.2. Future Work

The work presented herein presents the intra-activity and intra-subject variability for different sensor modalities, in particular Vision, EMG, IMUs and Goniometers. Additionally, the Separability Index, Reliability Index, Mean Semi-Principal Axis, and Desirability Scores were computed for each sensor modality. In general we found that Depth sensors had low variability, and by combining them with other sensors we obtained improved separability, repeatability, clustering, and overall performance relative to using any of the sensor modalities on their own or a combination of EMG and mechanical sensors alone. Additionally, though our analysis may have greater impact due to it being classifier and device agnostic, a specific prediction or control architecture may affect the performance based on each of these sensor modalities. In the future we aim to further consider the interaction between these sensory modalities, and develop an ideal system for combining features and sensors to yield reliable, consistent performance, and ultimately a generalizable forward predictor for use with a prosthetic leg or exoskeleton.

To ensure that our predictive system is robust we also plan to consider the effect of timing on our performance. In particular we will determine how the ability of depth to sense environmental changes substantially earlier than other sensor modalities can be incorporated with positive impacts on forward prediction. On a related note we will consider different approaches to sensor fusion that may capitalize on the specifics of our sensor modalities and. For instance, we can use a simple approach in which we combine all features prior to classification. Alternatively, it may be possible to use a weighted approach based on our understanding of the variability of each sensor modality developed in this work, and utilize a voting or Kalman filtering approach.

Once we integrate our online sensor-fusion based forward predictor into the prosthesis control system we will validate its performance with amputee subjects walking on a prosthesis, first in an offline control system, and later in an online controller. We will continue our testing with amputee subjects, over multiple days to determine the robustness of our prediction to different conditions. Importantly, we eventually aim to test our predictor outside of the laboratory environment, which will require wireless communication with the leg/controller, as well as validation that our system works comparably in different lighting conditions and environmental conditions. We will also consider the possibility of scanning a given environment (such as a home or office area, where someone may spend significant time) and using mapping to build an environment-specific forward predictor.

Finally, we think there are several exciting extensions to explore in future work. One of these is to incorporate environmental sensing into the mid/low level control of our device by modulating the specific trajectories or dynamics of the controller of the prosthetic leg, such as changing the toe clearance due to stair height, or increasing power to assist with steeper ramps. Additionally, since we have previously shown the ability to identify anticipated gait transitions several meters in advance [13], it may be possible to use this information to send feedback to user, or even to develop an augmented reality system to guide subjects during difficult transitions/gait events.

## 5. Conclusions

We collected able-bodied walking data to test how variability between subjects and activities differs across sensory modalities, in particular IMUs, EMG, goniometers and Vision-based sensing. Several Vision-based features were considered across ROIs of the field of view, including standard depth features, sagittal plane projections, optical flow features, normal features in the z-direction, and the normal-depth product. These feature sets were normalized to analyze intra-activity and intra-subject variability, and measures of separability, repeatability, clustering, and desirability between activities and subjects were also evaluated for each sensor modality. Ultimately, by combining the Vision features with EMG, IMU, and Goniometer feature sets we obtained the best performance across all metrics we computed. In the future we aim to utilize these feature sets in our development of a sensory-fusion driven forward prediction system for powered lower limb prostheses and exoskeletons.

## Abbreviations

The following abbreviations are used in this manuscript:

EMG	Electromyography
IMU	Inertial Measurement Unit
LW	Level Ground Walking
RA	Ramp Ascent
RD	Ramp Descent
SA	Stair Ascent
SD	Stair Descent
RANSAC	RANdom SAmpling Consensus
ROI	Region of Interest
RI	Repeatability Index
SI	Separability Index
MSA	Mean Semi-Principal Axis
DS	Desirability Score
HC	Heel Contact
TO	Toe Off
LDA	Linear Discriminant Analysis
PCA	Principal Component Analysis
AB	Able-Bodied
